# Predictors of Intensive Care Unit Outcomes Among Oncology Patients: A Retrospective Cohort Study at a Tertiary Referral Center in Saudi Arabia

**DOI:** 10.7759/cureus.103042

**Published:** 2026-02-05

**Authors:** Ahmed M Badheeb, Turki S Alayash, Udai O Alghanmi, Omar Alkhanbashi, Doaa A Eltohami, Safa Abdelrahman, Doaa Abdelmonem, Abdouh A Awad, Jobran Moshi, Esam Ben Yahya, Abdullah Abu Bakar

**Affiliations:** 1 Oncology, King Khalid Hospital, Najran, SAU; 2 Intensive Care Unit, Aseer Central Hospital, Khamis Mushait, SAU; 3 Internal Medicine, King Khalid Hospital, Najran, SAU; 4 Urology, King Khalid Hospital, Najran, SAU; 5 Anesthesia, King Khalid Hospital, Najran, SAU; 6 Medical Laboratory Technology, Jazan University, Jazan, SAU; 7 Ophthalmology, King Khalid Hospital, Najran, SAU

**Keywords:** cancer mortality, critical care oncology, intensive care unit, mechanical ventilation, neutropenia, organ dysfunction, prognostic factors, saudi arabia, sepsis

## Abstract

Background

Caring for critically ill cancer patients is challenging due to the complexity of malignancy combined with acute organ dysfunction. Despite an increasing cancer burden in Saudi Arabia, data on intensive care unit (ICU) outcomes, particularly in the Najran region, remain limited. This study aims to identify clinical characteristics, ICU treatments, and mortality predictors in this population.

Patients and methods

This retrospective cohort study included 105 adult patients with active solid or hematologic malignancies admitted to the ICU at King Khalid Hospital, Najran, between January 2014 and February 2023. Data, including demographics, cancer details, ICU admission causes, treatments, and outcomes, were collected. Multivariate logistic regression was used to assess mortality predictors, and Kaplan-Meier analysis compared survival by disease stage.

Results

The cohort had a mean age of 56.8 ± 16.5 years, with 57 females (54.3%). Advanced-stage disease was present in 84 patients (80.0%), with gastrointestinal cancers being the most common primary site (35, 33.3%). Cancer-related complications accounted for 77 ICU admissions (73.3%). The median Sequential Organ Failure Assessment (SOFA) score at admission was 1.0 (interquartile range (IQR) 0.0-3.0). ICU mortality was 40 patients (38.1%), primarily due to cancer progression. Independent mortality predictors included older age (adjusted odds ratio (aOR) 1.04 per year; 95% CI 1.01-1.06), advanced-stage disease (aOR 3.37; 95% CI 1.56-7.26), respiratory infections (aOR 2.19; 95% CI 1.04-4.59), and higher SOFA scores (aOR 1.70 per point; 95% CI 1.28-2.27). The predictive model demonstrated good discrimination (optimism-corrected area under the curve (AUC) = 0.82). Kaplan-Meier analysis showed significantly lower 14-day survival in advanced-stage patients (41%) compared to non-advanced stage patients (78%; log-rank p < 0.001).

Conclusions

This study highlights high ICU mortality among cancer patients at a tertiary center in southwestern Saudi Arabia, particularly those with older age, advanced-stage disease, respiratory infections, and higher SOFA scores. Early identification of these high-risk predictors is essential to guide clinical decision-making, enhance infection control, and facilitate timely palliative care integration. These findings provide valuable regional data to inform critical care strategies for oncology patients in similar settings.

## Introduction

Cancer remains a major global health challenge, significantly impacting mortality rates, healthcare systems, and socioeconomic stability of the country/countries. In 2022, the worldwide age-standardized incidence rate for all cancers combined was 196.9 per 100,000 population, with higher rates reported in men (212.6 per 100,000) compared to women (186.3 per 100,000) [1,2]. Excluding non-melanoma skin cancers, cancer continues to be a leading cause of death, accounting for 16.8% of all deaths (or mortality) and 22.8% of deaths from noncommunicable diseases (NCDs) [1]. Notably, it is a major contributor to premature mortality, responsible for 30.3% of NCD-related deaths among individuals aged 30-69 years and ranking among the top three causes of death within this age group in most countries worldwide [1]. Beyond these clinical impacts, cancer imposes substantial societal and economic burdens that vary by cancer type, geography, and gender [3].

Patients with cancer requiring intensive care unit (ICU) admission represent a particularly complex and vulnerable subgroup. Advances in oncological therapies and earlier diagnosis have increased the number of cancer patients who present with critical illness requiring ICU support. In Saudi Arabia, where cancer incidence is rising, understanding ICU outcomes and prognostic factors is essential but remains insufficiently studied [4,5]. Existing international literature identifies disease stage, performance status, and severity of organ dysfunction as key predictors of ICU survival in oncology patients [6,7]. However, the applicability of these findings to the Saudi population is uncertain, given differences in genetics, comorbidities, healthcare access, and treatment protocols.

Prognostication in ICU cancer patients is largely dependent on malignancy-specific characteristics and disease severity. Multiorgan failure at ICU admission is the strongest predictor of mortality, with risk escalating with the number of failing organ systems [5,8]. Patients with poor long-term prognoses are less likely to benefit from intensive care, underscoring the importance of accurate risk stratification. Certain malignancies, such as specific subtypes of acute myeloid leukemia and diffuse large B-cell lymphoma, show comparatively favorable outcomes following ICU admission, although disease status-new diagnosis versus relapse-remains a critical determinant of survival [8]. Despite routine use of cytogenetic and disease-specific risk scores, their prognostic value for ICU survival has not been fully validated, revealing an important gap in critical care oncology research [9].

At King Khalid Hospital’s Oncology Center, clinicians face frequent challenges in making ICU admission decisions for patients with solid and hematologic malignancies, often without robust local outcome data. This study aims to address this gap by evaluating the primary reasons for ICU admission, short-term mortality rates, and predictors of adverse outcomes in oncology patients. Additionally, it will assess patterns of organ dysfunction using standardized clinical scoring systems to better understand disease progression in this regional setting. By providing localized evidence, this research intends to enhance clinical decision-making, optimize ICU resource use, and promote timely integration of palliative care when appropriate, ultimately aiming to improve outcomes among cancer patients requiring critical care in Saudi Arabia’s healthcare system.

## Materials and methods

Study setting

This retrospective cohort study was conducted at King Khalid Hospital, a tertiary referral cancer center in Najran, Saudi Arabia, that manages over 40,000 patients annually [10]. The ICU is a dedicated 12-bed oncology unit providing specialized critical care for patients with active cancer. Standard ICU protocols include lung-protective ventilation (both invasive and non-invasive), renal replacement therapy, vasopressor support, and adherence to Surviving Sepsis Campaign guidelines for sepsis management. Hemodynamic monitoring routinely involves central venous pressure and arterial pressure measurement, supplemented by cardiac output assessment using LIDCO^TM^ technology. The hospital features a full-service emergency department and a 24-hour Clinical Assessment Unit responsible for rapid patient evaluation and ICU admission. ICU admission criteria prioritize patients with active malignancy undergoing treatment or presenting with cancer-related complications, including those admitted for intensive monitoring and non-escalatory support following shared decision-making, per institutional triage policies.

Study design and population

All adult patients (aged ≥18 years) with histologically confirmed active malignancies admitted to the medical ICU between January 2014 and February 2023 were included. Active malignancy was defined as patients receiving ongoing oncological treatment (chemotherapy, radiotherapy, immunotargeted therapy) or demonstrating evidence of disease progression within the last three months. Progression was defined per institutional standard using radiographic criteria (RECIST 1.1), unequivocal clinical deterioration, or rising tumor markers with correlative clinical findings. Patients transferred to other healthcare facilities post-ICU admission (n = 12) were excluded to ensure complete outcome data.

Data collection

Trained personnel extracted data retrospectively from electronic medical records using a standardized case report form. Collected variables comprised demographics (age, gender), oncological features (primary tumor site and cancer stage), prior treatment history, and ICU admission indications.

Cancer stage was classified as follows: for solid tumors, as metastatic (Stage IV) versus non-metastatic; for hematological malignancies, advanced-stage disease was defined using disease-specific systems (Ann Arbor staging for lymphomas and standard cytogenetic/molecular risk stratification for leukemias).

Severity of illness was assessed using the Sequential Organ Failure Assessment (SOFA) score within the first 24 hours of ICU stay [11]. This clinical tool is standardized; open-access scoring systems are widely used in critical care research and do not require specific licensure for academic, non-commercial use. 

ICU interventions recorded included vasopressor use, mechanical ventilation, and renal replacement therapy. Clinical outcomes comprised ICU and hospital mortality, and ICU length of stay. Performance status was assessed using the Eastern Cooperative Oncology Group (ECOG) scale within 48 hours prior to ICU admission. Neutropenia was defined as an absolute neutrophil count <500 cells/μL within 48 hours before or on ICU admission. A documented infection required a physician diagnosis supported by positive microbiological culture, radiological evidence, or a clinical/laboratory syndrome consistent with infection that prompted antimicrobial therapy. Comorbidities were documented from the medical record. A random 15% subset of records was independently verified by a second reviewer to ensure data accuracy.

Study outcomes

The primary outcome was ICU mortality. Secondary outcomes included hospital mortality, ICU length of stay, and identification of independent mortality predictors.

Statistical analysis

Statistical analyses were performed using IBM SPSS Statistics for Windows, Version 25.0 (released 2017, IBM Corp., Armonk, NY) and R software (version 4.3.0, R Foundation for Statistical Computing, Vienna, Austria, https://www.R-project.org/). Categorical variables were presented as frequencies and percentages, continuous variables as mean ± standard deviation (SD) or median with interquartile range (IQR). Group comparisons employed appropriate parametric or non-parametric tests.

Independent predictors of ICU mortality were identified via binary logistic regression (ENTER method). Covariates for the multivariate model were selected based on univariate analysis (p<0.20, a threshold chosen to be inclusive of potential confounders in this exploratory analysis) and clinical relevance. Multicollinearity was assessed using variance inflation factors (VIF < 5). Model performance was evaluated using the area under the receiver operating characteristic curve (AUC) and the Hosmer-Lemeshow goodness-of-fit test. Internal validation was performed using bootstrap resampling (1000 samples) to estimate the optimism-corrected AUC.

Missing data (<5% of observations, confined primarily to ECOG performance status and assumed to be missing completely at random) were handled by multiple imputation using the expectation-maximization algorithm with 10 imputed datasets. A sensitivity complete-case analysis yielded congruent results. Given the exploratory nature of this regional study and sample size constraints, a formal prospective power calculation was not performed. Statistical significance was set at two-tailed p < 0.05.

Ethical considerations

The study received approval from the Ethics Research Committee of King Khalid Hospital (approval number: H-11-N-136, July 16, 2025) and adhered to the Declaration of Helsinki. The Institutional Review Board waived the requirement for informed consent due to the retrospective design and minimal risk. Patient confidentiality was maintained through anonymization and secure data storage.

## Results

Patient demographics and clinical characteristics

The study cohort comprised 105 critically ill adult patients with active malignancy admitted to the ICU. Baseline characteristics are presented in Table 1. The mean age was 56.8 ± 16.5 years, with 57 (54.3%) female patients. Most admissions (92, 87.6%) originated from within the hospital network (internal referrals). Advanced-stage disease, defined as metastatic disease for solid tumors (stage IV) and advanced-stage (Ann Arbor stage III/IV) or high-risk disease for hematologic malignancies, was present in 84 patients (80.0%). The most common primary cancer sites were gastrointestinal (35, 33.3%), breast (13, 12.4%), and lymphoma (12, 11.4%). Specific hematologic malignancies included diffuse large B-cell lymphoma (n = 7), acute myeloid leukemia (n = 5), and other leukemia/lymphoma subtypes (n = 8). Diabetes mellitus was a prevalent comorbidity (70, 66.7%). At ICU admission, 98 patients (93.3%) were designated for full resuscitation. The seven patients with pre-ICU DNR orders were admitted for intensive monitoring and non-escalatory organ support during acute deterioration, following shared decision-making.

**Table 1:** Baseline demographics and clinical characteristics (N = 105) Data are presented as mean ± standard deviation (SD), median (interquartile range (IQR)), or number (%). *Advanced-stage disease encompasses metastatic solid tumors and advanced-stage/high-risk hematologic malignancies. †Lymphoma includes diffuse large B-cell lymphoma (n = 7) and other subtypes (n = 5). ‡Leukemia includes acute myeloid leukemia (n = 5) and other subtypes (n = 3). §Other solid tumors include ovarian, brain, sarcoma, and head and neck cancers. Abbreviations: ICU, intensive care unit; IQR, interquartile range

Characteristic	Value
Age, years	
Mean ± SD	56.8 ± 16.5
Median (IQR)	59.0 [45.0–72.0]
Age group, n (%)	
Young adults (18–39 years)	17 (16.2)
Adults (40–64 years)	47 (44.8)
Seniors (≥65 years)	41 (39.0)
Gender, n (%)	
Female	57 (54.3)
Male	48 (45.7)
Patient origin, n (%)	
Internal referral	92 (87.6)
External transfer	13 (12.4)
Disease stage, n (%)	
Advanced stage*	84 (80.0)
Non-advanced stage	21 (20.0)
Primary cancer site, n (%)	
Gastrointestinal*	35 (33.3)
Breast	13 (12.4)
Lymphoma†	12 (11.4)
Genitourinary	11 (10.5)
Leukemia‡	8 (7.6)
Lung	7 (6.7)
Other solid tumors§	19 (18.1)
Comorbidities, n (%)	
Diabetes mellitus	70 (66.7)
Hypertension	33 (31.4)
Chronic kidney disease	8 (7.6)
Pre-ICU code status, n (%)	
Full resuscitation	98 (93.3)
Do-not-resuscitate (DNR)	7 (6.7)

Indications for ICU admission and organ support

The primary reason for ICU admission was a direct complication of malignancy (77, 73.3%) (Table 2). A documented infection, defined by physician diagnosis supported by microbiological, radiological, or clinical/laboratory evidence, was present in 87 patients (82.9%), with respiratory infections being the most common (72, 82.8% of infected patients). Organ support was frequently required: 45 patients (42.9%) received invasive mechanical ventilation, and 40 (38.1%) required vasopressor or inotrope support. The median Sequential Organ Failure Assessment (SOFA) score at admission was 1.0 (IQR 0.0-3.0); this relatively low median score reflects that 28 patients (26.7%) had a score of 0, admitted primarily for close monitoring of evolving cancer complications or post-procedure care. The median ICU length of stay was four days (IQR 2-8).

**Table 2:** ICU admission characteristics and clinical interventions (N = 105) Data are presented as median (interquartile range (IQR)) or n (%). ‖Denominator (N) is the number of neutropenic patients (n = 10). Abbreviations: IQR, interquartile range; ANC, absolute neutrophil count; SOFA, Sequential Organ Failure Assessment; ICU, intensive care unit

Parameter	Value
Admission category, n (%)	
Directly cancer-related	77 (73.3)
Procedure-related	14 (13.3)
Other	14 (13.3)
Documented Infection, n (%)	87 (82.9)
Site of Infection (n=87), n (%)	
Respiratory infections	72 (82.8)
Urosepsis	3 (3.4)
Other	12 (13.8)
Neutropenia (ANC ≤1500/μL), n (%)	10 (9.5)
Severe neutropenia (ANC ≤500/μL), n/N (%) ‖	8/10 (80.0)
Active anticancer therapy, n (%)	56 (53.3)
Organ support, n (%)	
Invasive mechanical ventilation	45 (42.9)
Vasopressors/inotropes	40 (38.1)
Non-invasive ventilation	38 (36.2)
SOFA score, median (IQR)	1.0 (0.0–3.0)
ICU length of stay (days), median (IQR)	4.0 (2.0–8.0)

ICU outcomes and mortality

ICU and hospital mortality rates were 38.1% (n = 40) and 42.9% (n = 45), respectively (Table 3). The leading cause of death was progression of the underlying malignancy (27/40, 67.5%), as determined by the treating ICU team's clinical assessment documented in the death summary. A new do-not-resuscitate (DNR) order was instituted during the ICU stay for 17 patients (16.2%) at a median of three days (IQR 0-7) after admission. Notably, eight of these 17 patients (47.1%) died within 24 hours of the DNR order.

**Table 3:** Patient outcomes and mortality (N = 105) Data are presented as median (interquartile range (IQR)) or n (%). ¶Denominator is patients with new DNR orders (n = 17). Abbreviations: DNR, do-not-resuscitate; ICU, intensive care unit; IQR, interquartile range

Outcome	Value
Mortality, n (%)	
ICU mortality	40 (38.1)
Hospital mortality	45 (42.9)
Cause of death (n = 40), n (%)	
Cancer progression	27 (67.5)
Multi-organ failure	10 (25.0)
Treatment complications	3 (7.5)
New DNR orders in the ICU, n (%)	17 (16.2)
Time to DNR order (days), median (IQR)	3.0 (0.0–7.0)
Death <24 hours after DNR, n/N (%) ¶	8/17 (47.1)

Predictors of mortality

Univariate comparisons between survivors and non-survivors are shown in Table 4. Non-survivors were significantly older and more likely to have advanced-stage disease, respiratory infections, and higher SOFA scores.

**Table 4:** Univariate analysis of predictors for ICU mortality Abbreviations: SD, standard deviation; IQR, interquartile range; SOFA, Sequential Organ Failure Assessment; DNR, do-not-resuscitate; ICU, intensive care unit

Predictor	Survivors (n = 65)	Non-survivors (n = 40)	Test statistic; test name	p-value
Age (years), mean ± SD	53.8 ± 17.1	61.7 ± 14.2	t = 2.49; Independent samples t-test	0.014
Male gender, n (%)	26 (40.0)	22 (55.0)	χ² = 2.15; Chi-square test	0.142
Advanced-stage cancer, n (%)	39 (60.0)	38 (95.0)	χ² = 14.82; Chi-square test	<0.001
Respiratory infections, n (%)	37 (56.9)	35 (87.5)	χ² = 10.63; Chi-square test	0.001
SOFA score, median (IQR) [11]	0.0 [0.0–1.0]	2.0 [1.0–4.0]	U = 487.5; Mann-Whitney U test	<0.001
Diabetes mellitus, n (%)	40 (61.5)	30 (75.0)	χ² = 1.94; Chi-square test	0.164
Active therapy, n (%)	37 (56.9)	19 (47.5)	χ² = 0.87; Chi-square test	0.352
Pre-ICU DNR, n (%)	3 (4.6)	4 (10.0)	Fisher's exact test	0.283

In the multivariate logistic regression model (Table 5), four independent predictors of ICU mortality were identified: older age (adjusted odds ratio (aOR) 1.04 per year; 95% CI 1.01-1.06), advanced-stage disease (aOR 3.37; 95% CI 1.56-7.26), respiratory infections (aOR 2.19; 95% CI 1.04-4.59), and a higher SOFA score (aOR 1.70 per point; 95% CI 1.28-2.27). Internal validation via bootstrap resampling (1,000 samples) yielded an optimism-corrected AUC of 0.82. The model demonstrated adequate calibration (Hosmer-Lemeshow p = 0.79).

**Table 5:** Multivariate logistic regression model of ICU mortality predictors Model constructed via binary logistic regression (ENTER method). Data are presented as adjusted odds ratio (95% confidence interval). Model diagnostics: area under the curve (AUC) = 0.84; optimism-corrected AUC (bootstrap) = 0.82; Hosmer-Lemeshow goodness-of-fit χ² = 5.0 (p = 0.79) Abbreviations: CI, confidence interval; SOFA, Sequential Organ Failure Assessment

Predictor	Adjusted odds ratio (95% CI)	Wald statistic	p-value
Age (per year)	1.04 (1.01–1.06)	7.84	0.005
Male gender	1.66 (0.73–3.78)	1.47	0.226
Advanced-stage cancer	3.37 (1.56–7.26)	9.86	0.002
Respiratory infections	2.19 (1.04–4.59)	4.30	0.038
SOFA score (per point) [11]	1.70 (1.28–2.27)	14.21	<0.001
Diabetes mellitus	1.46 (0.63–3.38)	0.76	0.382
Active anticancer therapy	0.65 (0.29–1.46)	1.10	0.295

Survival analysis

**Figure 1:**
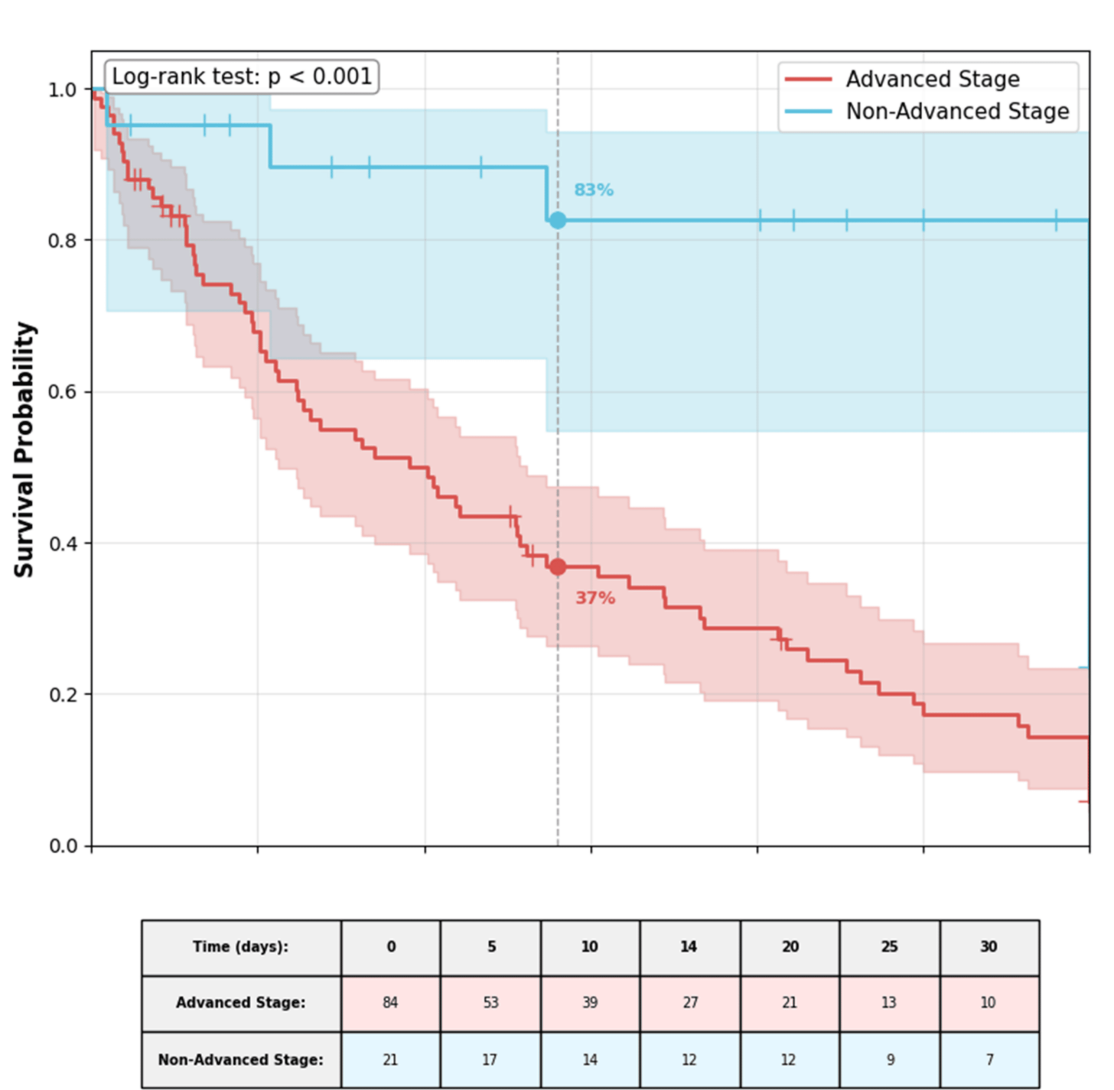
Kaplan-Meier survival curves for ICU survival stratified by disease stage. Patients with advanced-stage disease (encompassing metastatic solid tumors and advanced hematologic malignancies) exhibited significantly lower survival probability compared to patients with non-advanced stage disease (log-rank p < 0.001). The shaded areas represent 95% confidence intervals. The dashed vertical line indicates the 14-day time point with corresponding survival probabilities annotated.

Kaplan-Meier analysis of ICU survival showed significantly worse outcomes for patients with advanced-stage disease compared to those without (log-rank p < 0.001; Figure 1). The estimated 14-day ICU survival probability was 78% (95% CI 65-88%) for non-advanced stage patients versus 41% (95% CI 31-53%) for advanced-stage patients.

## Discussion

This single-institution study of cancer patients who were admitted to the ICU showed a mortality rate of 38.1%. Furthermore, it identified advanced-stage malignancies and respiratory tract infections at ICU admission as independent predictors of mortality among critically ill cancer patients. These factors, along with older age and higher SOFA score, were further recognized as statistically significant predictors of ICU mortality in multivariate analysis.

Overall, studies evaluating mortality among critically ill cancer patients demonstrate notable variability. In a scoping review by Cordu et al, mortality ranged from 8% to 72%, with a weighted mean of approximately 41% [4]. In another multicenter study, cancer patients with sepsis had a pooled ICU mortality of 48% [5]. Lower mortality trends were reported in the South American region, with a mortality rate of 21%. However, these pooled analyses included patients admitted for non-medical reasons, including scheduled surgeries, who had a mortality rate of 6%, compared with patients admitted to medical ICUs, who experienced mortality rates of 44% [6]. Studies of ICU mortality among cancer patients in Saudi Arabia are limited; a few reports showed mortality rates of 52% and 61% among solid and hematologic malignancies, respectively [7]. A more recent study by Al Khamis et al showed a 28-day mortality rate of 48% [8]. This variation in mortality is primarily related to heterogeneity in patient characteristics, cancer sites, disease stages, and levels of ICU care. It likely also reflects differences in organ support requirements and patient functional status.

In our study, gastrointestinal malignancies were most prevalent (33.3%), which aligns with the high regional incidence of GI cancers, primarily colorectal cancer [9]. Indeed, population-based studies have shown that GI cancers account for more than 41% of cancer-related ICU admissions [10,11]. This pattern is likely driven by cancer-related complications such as perforation, obstruction, sepsis, or bleeding that necessitate ICU admission. Furthermore, these patients are often admitted for postoperative monitoring [12-14].

The mean age in our cohort was 56.8 years, somewhat lower than that reported in a prior multicenter Chinese study showing a mean age of 63.2 years [15]. Nevertheless, prior studies demonstrate a wide ICU admission age range, from 25 to 81 years [16]. As noted earlier, heterogeneity in study design, cancer subtypes, and ICU admission indications limits cross-study comparisons. Notably, about two-thirds of our cohort had diabetes mellitus, a prevalence markedly higher than the global average of 8-32% [17]. This is likely related to the high age-standardized prevalence of diabetes in Saudi Arabia and the Middle Eastern region. In addition, our cohort included older patients, who have nearly double the diabetes risk compared with younger groups; Bahijri et al reported a prevalence exceeding 40% in individuals older than 40 years [18]. Approximately one-third of our patients had hypertension, which is generally consistent with international reports showing a prevalence near 38% among cancer patients [19]. Indeed, prevalence may rise substantially among patients receiving certain chemotherapeutic agents, reaching rates as high as 80% [20]. Chronic kidney disease was less common in our cohort (7.6%). Reported CKD prevalence varies widely by cancer subtype, from 2.5% in brain neoplasms to up to 50% in renal carcinomas [21]. Nevertheless, diabetes, hypertension, and chronic kidney disease did not differ significantly between survivors and non-survivors in our study.

Respiratory infections were the most common infection source in our cohort, followed by urosepsis. Notably, respiratory failure is a major indication for ICU admission among cancer patients, most often driven by infectious etiologies and associated with increased mortality risk [22,23]. In one study, pneumonia accounted for approximately 30% of ICU admissions among cancer patients, with nearly half requiring mechanical ventilation [24]. In a single-centre experience from Jordan, respiratory failure was also the most common indication for ICU admission, although the underlying etiologies were not specified [25]. Overall, compared with non-cancer patients, those with malignancy experience nearly double the mortality, with reported hospital mortality rates approaching 64.9% [4,26].

Another important finding in our study was the increased mortality associated with advanced cancer status, with more than a threefold increase in adjusted mortality odds. Similar findings have been reported in several prior studies linking cancer progression with worse ICU outcomes [27,28]. However, these observations are not universal. McGrath et al reported modest but notable outcome improvements among ICU patients with advanced malignancy in the last decade [29]. The authors emphasized that much of the evidence suggesting worse outcomes is limited by retrospective design and patient heterogeneity. While further studies are needed to clarify this relationship, such designs may be ethically challenging, as they could lead to premature withholding of care from patients who may benefit from ICU-level support.

The SOFA score was also a significant mortality predictor in our study. Indeed, SOFA is widely accepted as a prognostic tool, with higher scores associated with worse outcomes in sepsis [30]. Among cancer patients, SOFA is a sensitive and accurate method for identifying and risk-stratifying patients with suspected infection, both inside and outside ICU settings [30-32]. However, it is important to recognize that SOFA is neither a diagnostic nor a management tool, as emphasized by critical care societies [33]. Thus, a normal initial SOFA score should not be interpreted as the absence of infection. In our cohort, despite a median SOFA score of only 1.0, mortality remained high at 38.1%. Prior reviews show that repeated SOFA assessments improve mortality prediction independently of initial values [34]. Our retrospective design did not permit reliable sequential SOFA measurement, which may partly limit the generalizability of our findings.

The timing of DNR orders in our cohort warrants critical examination. The observation that 47.1% of patients with new DNR orders died within 24 hours suggests that goals-of-care discussions and palliative care integration often occurred at a very advanced stage of critical illness. This pattern highlights potential missed opportunities for earlier conversations about care preferences and treatment limitations. While prognostication in critically ill cancer patients is challenging, these findings underscore the need for systematic approaches to facilitate timely palliative care referral and goals-of-care discussions, particularly for patients identified as high-risk by predictive models such as ours.

Clinical implications

Early identification of high-risk patients using the identified predictors, i.e., age, disease stage, SOFA score, and presence of respiratory infections, can guide clinical decision-making. This facilitates timely aggressive intervention for those who may benefit and prompts early palliative care involvement for others. Our predictive model, with an optimism-corrected AUC of 0.82, offers a validated tool for risk stratification in similar settings. The high burden of infectious complications mandates rigorous preventive measures and prompt, targeted antimicrobial therapy. These findings provide a data-driven foundation for developing local, multidisciplinary protocols to optimize the care of critically ill oncology patients in this region.

Study limitations

This study has several limitations that should be considered when interpreting the findings. First, its retrospective and single-center design may limit the generalizability of results to broader populations and healthcare settings. Second, the relatively modest sample size, while comparable to similar oncology ICU studies, constrains statistical power, limits the precision of our estimates as reflected in the confidence intervals, and precludes detailed subgroup analyses. Third, the classification of "advanced-stage disease," while clarified, presents a methodological challenge in creating a uniform prognostic category across solid and hematologic malignancies.

Fourth, some relevant clinical data, including detailed oncologic treatment regimens and biomarkers, were not uniformly available. Potential biases include the exclusion of transferred patients (n = 12), which may affect outcome assessment, and the long study period over which ICU and oncological practices may have evolved. Finally, the attribution of cause of death was based on clinical assessment, which may involve subjectivity and overlap between categories such as cancer progression and multi-organ failure. Despite these constraints, the study provides valuable regional insights and establishes a foundation for future prospective, multicenter research.

## Conclusions

Critically ill cancer patients admitted to the ICU at King Khalid Hospital, Najran, demonstrated a substantial mortality risk. Independent predictors of poor outcome included older age, advanced-stage disease (encompassing metastatic solid tumors and advanced hematologic malignancies), respiratory infections, and higher SOFA score. These results emphasize the need for early, data-driven risk stratification to guide clinical decision-making, prompt infection management, and facilitate the timely integration of palliative care services, particularly given the observed pattern of late goals-of-care discussions.

The development of local, multidisciplinary protocols for ICU triage and management is encouraged. The regional evidence presented herein can inform critical care strategies for oncology patients in similar settings; however, these findings require validation in larger, prospective multicenter studies. Ultimately, a patient-centered approach that balances aggressive support with realistic prognostication is essential to improve outcomes for this vulnerable population.

## References

[1] Bray F, Laversanne M, Sung H, Ferlay J, Siegel RL, Soerjomataram I, Jemal A (2024). Global cancer statistics 2022: GLOBOCAN estimates of incidence and mortality worldwide for 36 cancers in 185 countries. CA Cancer J Clin.

[2] GBD 2021 Diseases and Injuries Collaborators (2024). Global incidence, prevalence, years lived with disability (YLDs), disability-adjusted life-years (DALYs), and healthy life expectancy (HALE) for 371 diseases and injuries in 204 countries and territories and 811 subnational locations, 1990-2021: a systematic analysis for the Global Burden of Disease Study 2021. Lancet.

[3] van der Zee EN, Benoit DD, Hazenbroek M, Bakker J, Kompanje EJ, Kusadasi N, Epker JL (2021). Outcome of cancer patients considered for intensive care unit admission in two university hospitals in the Netherlands: the danger of delayed ICU admissions and off-hour triage decisions. Ann Intensive Care.

[4] Codru IR, Vecerzan L (2025). When and for whom does intensive care unit admission change the prognosis in oncology?-a scoping review. Cancers (Basel).

[5] Nazer L, Lopez-Olivo MA, Cuenca JA (2022). All-cause mortality in cancer patients treated for sepsis in intensive care units: a systematic review and meta-analysis. Support Care Cancer.

[6] Soares M, Caruso P, Silva E (2010). Characteristics and outcomes of patients with cancer requiring admission to intensive care units: a prospective multicenter study. Crit Care Med.

[7] S Lababidi HM, Alajlani A, Alasmari A, Alshammeri W, Suwayyid WK, Bahnassy AA (2019). The characteristics and outcomes of oncology patients in intensive care unit in a tertiary care hospital in Saudi Arabia. Saudi Critical Care Journal.

[8] AlSaied G, Lababidi H, AlHawdar T, AlZahrani S, AlMotairi A, AlMaani M (2024). Outcome of cancer patients with an unplanned intensive care unit admission: predictors of mortality and long-term survival. Saudi J Med Med Sci.

[9] Alessy SA, Al-Zahrani A, Alhomoud S (2025). Towards a comprehensive cancer control policy in Saudi Arabia. Lancet Oncol.

[10] Puxty K, McLoone P, Quasim T, Sloan B, Kinsella J, Morrison DS (2015). Risk of critical illness among patients with solid cancers: a population-based observational study. JAMA Oncol.

[11] Liu W, Zhou D, Zhang L (2024). Characteristics and outcomes of cancer patients admitted to intensive care units in cancer specialized hospitals in China. J Cancer Res Clin Oncol.

[12] (2025). Acute presentations of colorectal cancer: an international prospective snapshot on management and outcomes. World J Surg.

[13] Pajola M, Fugazzola P, Cobianchi L, Frassini S, Ghaly A, Bianchi C, Ansaloni L (2024). Surgical emergencies in rectal cancer: a narrative review. J Clin Med.

[14] Epstein AS, Yang A, Colbert LE, Voigt LP, Meadows J, Goldberg JI, Saltz LB (2020). Outcomes of ICU admission of patients with progressive metastatic gastrointestinal cancer. J Intensive Care Med.

[15] Wei M, Huang M, Duan Y (2023). Prognostic and risk factor analysis of cancer patients after unplanned ICU admission: a real-world multicenter study. Sci Rep.

[16] Hautecloque-Raysz S, Thananayagam MA, Martignene N, Deley M-CL, Penel N, Carnot A (2021). Overall survival (OS) and prognostic factors (PF) of patients (pts) with metastatic solid tumors admitted in intensive care unit (ICU). J Clin Oncol.

[17] Ose DJ, Viskochil R, Holowatyj AN (2021). Understanding the prevalence of prediabetes and diabetes in patients with cancer in clinical practice: a real-world cohort study. J Natl Compr Canc Netw.

[18] Bahijri SM, Jambi HA, Al Raddadi RM, Ferns G, Tuomilehto J (2016). The prevalence of diabetes and prediabetes in the adult population of Jeddah, Saudi Arabia--a community-based survey. PLoS One.

[19] Piccirillo JF, Tierney RM, Costas I, Grove L, Spitznagel EL Jr (2004). Prognostic importance of comorbidity in a hospital-based cancer registry. JAMA.

[20] Bloom MW, Vo JB, Rodgers JE (2025). Cardio-oncology and heart failure: a scientific statement from the Heart Failure Society of America. J Card Fail.

[21] Ciorcan M, Chisavu L, Mihaescu A (2022). Chronic kidney disease in cancer patients, the analysis of a large oncology database from Eastern Europe. PLoS One.

[22] Azoulay E, Mokart D, Kouatchet A, Demoule A, Lemiale V (2019). Acute respiratory failure in immunocompromised adults. Lancet Respir Med.

[23] Benguerfi S, Dumas G, Soares M (2023). Etiologies and outcome of patients with solid tumors admitted to ICU with acute respiratory failure: a secondary analysis of the EFRAIM Study. Respir Care.

[24] Maschmeyer G, Bertschat FL, Moesta KT (2003). Outcome analysis of 189 consecutive cancer patients referred to the intensive care unit as emergencies during a 2-year period. Eur J Cancer.

[25] Hawari FI, Nazer LH, Addassi A, Rimawi D, Jamal K (2016). Predictors of ICU admission in patients with cancer and the related characteristics and outcomes: a 5-year registry-based study. Crit Care Med.

[26] Heybati K, Deng J, Bhandarkar A (2024). Outcomes of acute respiratory failure in patients with cancer in the United States. Mayo Clin Proc.

[27] Bosch-Compte R, Visa L, Rios A, Duran X, Fernández-Real M, Gomariz-Vilaldach G, Masclans JR (2023). Prognostic factors in oncological patients with solid tumours requiring intensive care unit admission. Oncol Lett.

[28] Fisher R, Dangoisse C, Crichton S, Whiteley C, Camporota L, Beale R, Ostermann M (2016). Short-term and medium-term survival of critically ill patients with solid tumours admitted to the intensive care unit: a retrospective analysis. BMJ Open.

[29] McGrath S, Chatterjee F, Whiteley C, Ostermann M (2010). ICU and 6-month outcome of oncology patients in the intensive care unit. QJM.

[30] Seymour CW, Liu VX, Iwashyna TJ (2016). Assessment of clinical criteria for sepsis: for the Third International Consensus Definitions for Sepsis and Septic Shock (Sepsis-3). JAMA.

[31] Finkelsztein EJ, Jones DS, Ma KC (2017). Comparison of qSOFA and SIRS for predicting adverse outcomes of patients with suspicion of sepsis outside the intensive care unit. Crit Care.

[32] Costa RT, Nassar AP Jr, Caruso P (2018). Accuracy of SOFA, qSOFA, and SIRS scores for mortality in cancer patients admitted to an intensive care unit with suspected infection. J Crit Care.

[33] Singer M, Deutschman CS, Seymour CW (2016). The Third International Consensus Definitions for Sepsis and Septic Shock (Sepsis-3). JAMA.

[34] Ferreira FL, Bota DP, Bross A, Mélot C, Vincent JL (2001). Serial evaluation of the SOFA score to predict outcome in critically ill patients. JAMA.

